# Effect of egualen sodium hydrate on small-intestinal mucosal damage induced by low-dose aspirin: a prospective randomized clinical trial

**DOI:** 10.3164/jcbn.17-46

**Published:** 2018-01-11

**Authors:** Munetaka Iguchi, Kazuki Kakimoto, Takanori Kuramoto, Kei Nakazawa, Minori Kubota, Yuki Hirata, Kaori Fujiwara, Satoshi Harada, Taisuke Sakanaka, Kazuhiro Ota, Shoko Edogawa, Yuichi Kojima, Sadaharu Nouda, Toshihiko Okada, Ken Kawakami, Toshihisa Takeuchi, Takuya Inoue, Kazuhide Higuchi

**Affiliations:** 1 Second Department of Internal Medicine, Osaka Medical College, 2-7 Daigakumachi, Takatsuki, Osaka 569-8686, Japan

**Keywords:** NSAIDs, small intestinal injury, video capsule endoscopy, egualen sodium hydrate, aspirin

## Abstract

Low-dose aspirin, which is widely used to reduce the risk of cardio- and cerebrovascular thrombosis, often induces gastroenteropathy by increasing the permeability of the mucosa. However, therapeutic strategies for patients with low-dose aspirin-induced small intestinal injury have not been determined. We evaluated the preventative effect of egualen sodium hydrate, a gastro-protective agent that suppresses indomethacin-induced small-intestinal damage in rats, against small-intestinal mucosal damage induced by low-dose aspirin in healthy adult male volunteers. Participants were randomly allocated to receive aspirin 100 mg/kg daily (control group, *n* = 10) or aspirin 100 mg/kg plus egualen sodium 30 mg daily (egualen sodium group, *n* = 10). Small intestinal mucosal injury was evaluated by capsule endoscopy two weeks after initiation of drug administration. Fecal analyses (occult blood test, immunochemical test, transferrin measurement and calprotectin measurement) were carried out before and after treatment. Egualen sodium significantly suppressed the total number of small intestinal injuries detected by capsule endoscopy and the positive ratio for the fecal occult blood test. Daily use of 30 mg of egualen sodium showed a preventative effect on low-dose aspirin-induced small intestinal injury. Since acid suppression therapy was reported to exacerbate NSAIDs-induced enteropathy via dysbiosis, egualen sodium may be useful for patients treated with low-dose aspirin.

## Introduction

Nonsteroidal anti-inflammatory drugs (NSAIDs) are widely used to treat chronic pain because of their anti-inflammatory and analgesic effects; however, they are commonly known to induce a variety of lesions in the gastric mucosa, such as erosions, hemorrhagic lesions and gastric ulcers.^(1,2)^ Recent advances in digestive tract endoscopy, including the development of wireless video capsule endoscopy and double-balloon enteroscopy, have facilitated detailed examinations of the entire digestive tract.^(3–6)^ As a result, it has been shown that NSAIDs induce not only gastric, but also small/large intestinal mucosal injuries, such as erosion and ulcers.^(7,8)^ Low-dose aspirin is also widely used to reduce the risk of cardio- and cerebrovascular thrombosis.^(9,10)^ Although the severity of mucosal injuries is milder in patients receiving low-dose aspirin compared with that in patients receiving non-aspirin NSAIDs,^(11)^ low-dose aspirin also often induces gastroenteropathy by increasing the permeability of the mucosa.^(12,13)^

Various drugs, such as rebamipide, misoprostol, sulfasalazine, and antibiotics, have been considered candidates for NSAID-induced intestinal injury.^(14,15)^ It has been reported that combined treatment with rebamipide and the proton pump inhibitor (PPI) omeprazole prevented NSAID-induced gastrointestinal symptoms, especially lower gastrointestinal symptoms.^(16)^ Although potent anti-secretory therapy can prevent NSAID-induced foregut mucosal injury, the usefulness of PPIs remains controversial.^(17)^ Thus far, therapeutic strategies for the treatment of patients with NSAID-induced small intestinal mucosal injury have not been determined.

Water-soluble azulene (sodium guaiazulene-3-sulfonate) has been used for the treatment of gastric ulcer and gastritis.^(18)^ Egualen sodium, a stable azulene derivative, is also widely used for the treatment of gastric ulcer.^(18)^ Although its actual mechanisms of action have not been fully elucidated, the mechanisms responsible for the antiulcer effect of egualen sodium are reported to be increased mucosal blood flow,^(19)^ prevention of vascular injury, prevention of decreases in endogenous sulfhydryl (SH) compounds^(20)^ and blockade of thromboxane (TX) A2 receptors.^(21)^ Regarding the protective effect of egualen sodium on small intestinal mucosal injury, Amagase *et al.*^(22)^ showed that the prophylactic effect of egualen sodium on small intestinal damage in rats occurred through its characteristic pharmacological properties. However, thus far, no study has evaluated the protective effect of egualen sodium on small intestinal injury in humans.

## Materials and Methods

### Subjects

Subjects eligible for inclusion were healthy adults who 1) were between 20 and 79 years of age when consent was obtained, 2) had freely given their fully informed consent based on their full understanding of the study and 3) had taken no medication during the one-month period before the start of the study. The exclusion criteria were 1) a history of peptic ulcer or gastrointestinal bleeding, 2) significant hepatic, renal, heart, or respiratory disease, 3) a history of gastrointestinal surgery other than appendectomy, 4) oral use or planned oral use of a drug other than an antiulcer drug, 5) alcohol or chemical dependency, 6) a history of intestinal obstruction or suspected gastrointestinal obstruction on other tests, 7) a lack of consent to the surgery required if the capsule endoscope was retained in the body and 8) determination by the investigator, at his or her discretion, that a subject was ineligible for participation in the study for any other reason.

### Study protocol

**Fig. 1:**
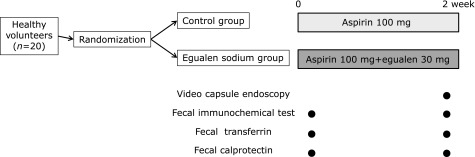
Study protocol.

This was a randomized controlled video capsule endoscopy trial on the efficacy of egualen sodium hydrate to prevent small intestinal injury induced by low-dose aspirin in healthy subjects. Twenty healthy adult male volunteers (20 years or older) were randomly divided into two groups. In the egualen sodium group, egualen sodium hydrate 30 mg/day and aspirin 100 mg/day were administered orally for two weeks (Fig. 1). In the control group, aspirin 100 mg/day were administered orally for two weeks. The dose of egualen sodium hydrate was based on the dose approved by the Japanese Ministry of Health and Welfare. Small intestinal mucosal injury was evaluated before and two weeks after the start of drug administration with capsule endoscopy. According to our previous report, fecal occult blood, transferrin, and calprotectin were also evaluated before and two weeks after the start of drug administration.^(23)^

### Ethics

This study was approved by the Ethics Review Committee of Osaka Medical College, and informed consent was obtained from all patients. Approval number is 730-01.

### Capsule endoscopy

**Fig. 2:**
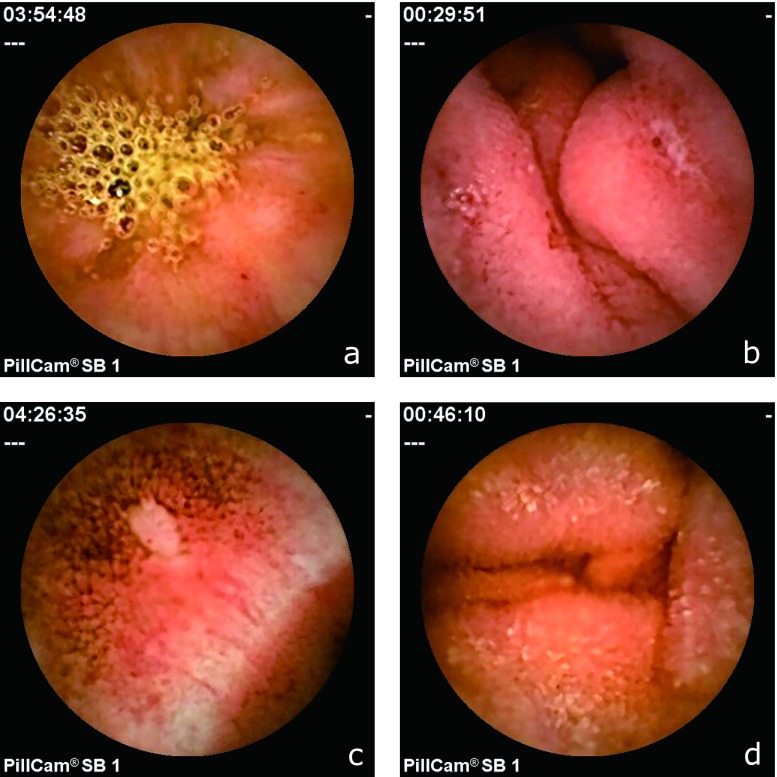
Typical findings of capsule endoscopy in the small intestine. a: erythema, b: erosion, c: ulcer, d: edema.

Video capsule endoscopy was performed with the PillCam SB1 (Given Imaging, Ltd., Yoqneam, Israel). For pretreatment prior to capsule endoscopy, as we previously described, the subjects were fasted for 12 h before swallowing the capsule (water was permitted).^(24)^ The subjects received 1 L of polyethylene glycol solution (Niflec^®^, Ajinomoto Pharma Co., Ltd., Tokyo, Japan) containing 200 mg of dimethylpolysiloxane (Baros^®^, Horii Pharmaceutical Ind., Ltd., Osaka, Japan) 3 h before the examination. Data were collected for up to 8 h after capsule ingestion. Then, the sensory array and recording device were removed. The capsule digital image stream was reviewed, and images of the small intestine were independently evaluated by two digestive endoscopists (M.I. and T.K.) who were not informed of the subjects’ background and have experienced in performing roughly 100 capsule endoscopy thus far. Small intestinal mucosal injury was assessed based on the number of findings with respect to four types of injury, as follows: erythema, erosion, ulcer and edema (Fig. 2). Erythema was defined as a red region with a border extending from the peripheral normal mucosa, erosion was defined as a defect of the normal lustrous mucosa, ulcer was defined as mucosal defects covered with white moss, and edema was defined as villous swelling based on the classification reported by Fujimori *et al.*^(25)^ and Niwa *et al.*,^(26)^ with slight modifications.

### Noninvasive tests of intestinal damage

Subjects collected a stool sample for determination of fecal calprotectin as a measure of intestinal inflammation at baseline and the final visit. Stools were frozen within 12 h of receipt and stored at −20°C for subsequent analysis with an enzyme-linked immunosorbent assay kit (Immundiagnostik, Bensheim, Germany) as previously described. Results are expressed as micrograms of calprotectin per gram of stool, and a cutoff value of 50 µg/g stool was used, as recommended by the manufacturer. The fecal calprotectin value has the problem of variation, so we decided to use the -fold increase after treatment, when the calprotectin concentration before treatment was set to 1. Before and after the study, fecal occult blood was assessed with the tetramethylbenzidine and guaiac tests with occult fecal blood slide kits from Shionogi Pharma (Osaka, Japan). In both tests, the color intensity of the oxidation product was assigned to one of four categories, 3+, 2+, + or −. The hemoglobin and transferrin antibody tests for occult fecal blood were performed with an OCMicro analyzer (Eiken, Tokyo, Japan). Generally, fecal occult blood is influenced by the intake of meat, fish, bright red, green or yellow vegetables, and so on. Therefore, we explained to our subjects how these foods affect the results of the occult blood tests (the tetramethylbenzidine test and the guaiac test), and suggested that they pay attention to their food intake during the 4-day period prior to the examination date.

### Randomization

A coordinator performed a simple fixed-allocation randomization using a block-randomization scheme. Random numbers were generated by SAS (SAS Institute, Cary, NC).

### Statistical analysis

For continuous or categorical variables, the statistical significance of differences between groups was determined with the *t* test. For binary variables, the statistical significance of differences between groups was determined with the chi-square test. All reported *p* values are two-sided, and values of less than 0.05 were considered to indicate statistically significant differences. All calculations were made with the Statview system (SAS Institute, Cary, NC).

## Results

### Baseline characteristics

This study was conducted prospectively from April to September 2012 at Osaka Medical College Hospital. The 20 healthy volunteers were randomly assigned to either the control group or the egualen sodium group and underwent capsule endoscopy twice within two weeks. One volunteer assigned to the control group did not complete the study because the capsule did not pass the entire length of the small intestine before the battery was out. Therefore, the small intestinal lesions of nine volunteers in the control group and ten volunteers in the egualen sodium group were evaluated. The characteristics of the subjects, including age, sex, weight, height and small bowel transit time were not significantly different between groups (Table 1). With regard to the noninvasive tests of intestinal damage, fecal immunochemical test levels, fecal transferrin levels, and fecal calprotectin levels were 25.0 ± 9.3 ng/ml, 8.4 ± 4.9 ng/ml and 5,294.7 ± 5,489.2 µg/g, respectively, in the control group and 134.8 ± 187.9 ng/ml, 34.1 ± 86.8 ng/ml and 9,319.2 ± 16,519.6 µg/g, respectively, in the egualen sodium group. The results of the noninvasive tests before the initiation of treatment were not significantly different between groups.

### Evaluation of small intestinal lesions

**Fig. 3:**
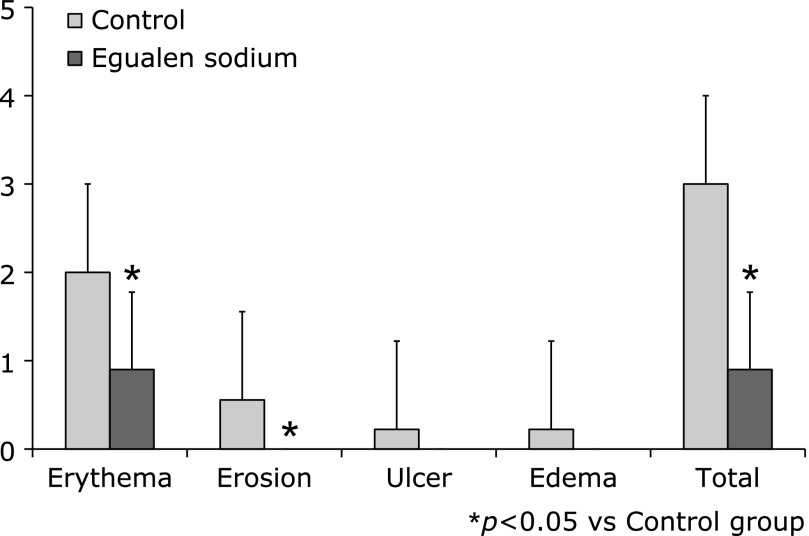
Number of small intestinal lesions in each group. The total incidence of small intestinal injuries, erythema and erosions was significantly suppressed in the egualen sodium group.

As shown in Fig. 2, several capsule endoscopic findings of typical small intestinal mucosal injuries related to the two-week administration of aspirin were observed. The total number of intestinal lesions was significantly reduced in the egualen sodium group compared to the control group (Table 2). No ulcerous lesions, erosions, or edematous lesions were detected in the egualen sodium group (Fig. 3). The incidence of erythema, erosions, ulcers and edema in the control group vs the egualen sodium group was 1.80 ± 0.92 vs 0.90 ± 0.88 (*p* = 0.038), 0.60 ± 0.70 vs 0.0 ± 0.0 (*p* = 0.014), 0.20 ± 0.42 vs 0.0 ± 0.0 (*p* = 0.15) and 0.20 ± 0.42 vs 0.0 ± 0.0 (*p* = 0.15), respectively. Concordance rate between two endoscopist’s diagnoses was over 90%.

### Fecal immunochemical test levels, fecal transferrin levels, and fecal calprotectin levels

Two weeks after the treatment, the noninvasive tests showed that fecal immunochemical test levels, fecal transferrin levels, fecal calprotectin levels and the -fold increase of fecal calprotectin were 56.6 ± 91.2 ng/ml, 7.4 ± 3.7 ng/ml, 13,949.8 ± 17,402.8 µg/g and 5.3 ± 8.5, respectively in the control group and 21.8 ± 3.2 ng/ml, 6.7 ± 3.0 ng/ml, 15,168.4 ± 26,907.5 µg/g and 3.2 ± 5.7, respectively, in the egualen sodium group (Table 3). Although there was no significant difference, fecal immunochemical test levels, fecal transferrin levels, and fecal calprotectin levels (-fold increase of fecal calprotectin) in the egualen sodium group were lower than those in the control group. The positive reaction in fecal occult blood test was significantly lower in the egualen sodium group compared to the control group.

## Discussion

To our knowledge, this is the first report to evaluate the protective effect of egualen sodium on small intestinal injury in human subjects and to show that the daily administration of egualen sodium significantly decreased the number of small intestinal injuries identified by capsule endoscopy. Daily administration of egualen sodium also significantly decreased the prevalence of positive fecal occult blood tests in the human volunteers who received low-dose aspirin.

Previous studies have shown a prophylactic effect of egualen sodium in various models of damage in the stomach and duodenum; thus, egualen sodium has been used for the treatment of gastritis and gastric ulcers.^(18–21)^ Thus far, there is one study evaluating the effect of egualen sodium on small intestinal injury.^(22)^ Amagese *et al.*^(22)^ evaluated the protective effect of egualen sodium on small intestinal injury in rats and clarified that egualen sodium exerts a mucosal protective effect by stimulation of mucus secretion, inhibition of bacterial invasion, and inhibition of iNOS expression. Before conducting the present study, we also preliminarily evaluated the effect of egualen sodium on NSAID-induced small intestinal injury in rats. Small intestinal injury was induced by a single administration of indomethacin (10 mg/kg i.g.) and was measured 24 h after the administration of indomethacin. Egualen sodium was administered 30 min before and 6 h after the administration of indomethacin. As a result, the oral administration of egualen sodium was equally distributed to the entire small intestine, and 100 mg/kg of egualen sodium significantly prevented the NSAID-induced small intestinal ulcers. Although these data were not published yet, we have confirmed the protective effect of egualen sodium on NSAID-induced small intestinal injury in rats with the approval by the Ethics Review Committee of Osaka Medical college before conducting the present study in humans.^(27)^

Aspirin induces vascular permeability and subsequent mucosal injury.^(28)^ However, non-aspirin NSAIDs, such as indomethacin, but not aspirin, are generally used in the experiment for the treatment of NSAIDs-induced small intestinal injury because non-aspirin NSAIDs can easier induce small intestinal ulcers than aspirin.^(29–31)^ In human studies, we usually use diclofenac sodium to induce small intestinal injury and omeprazole, which was used to prevent gastroduodenal adverse effects.^(32,33)^ Indeed, the number of patients who use low-dose aspirin for the prevention of vascular thrombosis has increased recently. Therefore, we conducted the present study using low-dose aspirin to induce small intestinal injury, although the use of diclofenac sodium might more clarify the results than aspirin.

Generally, anti-acid agents, such as histamine receptor type 2 (H2) blockers and PPIs, are administered to patients who need anti-platelet treatment to prevent the gastrointestinal bleeding induced by aspirin.^(34,35)^ PPI are reported to suppress gastrointestinal symptoms and recurrent peptic ulcers/erosions in patients treated with aspirin.^(35)^ With regard to small intestinal bleeding, gut microbiota may play a major role in the pathophysiology of NSAID-induced enteropathy, and PPIs have been proved to cause dysbiosis in the intestine in an experimental model.^(17)^ Therefore, chronic acid suppression using PPIs and H2 blockers may exacerbate NSAID-induced small intestinal injury even though they suppress gastric and duodenal ulcers/erosions. Indeed, some reports describing the risk of PPIs on the small intestinal injury in human have been recently published although the interaction between NSAIDs and PPIs use for small intestinal bleeding have been still under controversial.^(36–39)^ Therefore, we consider that mucosal protection, which is independent of acid-suppression, will be necessary to prevent NSAID-induced enteropathy for patients receiving long-term treatment with aspirin.

Our study has some limitations. First, this study did not involve placebo and so this was not double blinded study. Second, sample size was not determined before the conductance of this study and that was small. Third, small intestinal lesions before the administration of aspirin were not evaluated. Because the administration of egualen sodium significantly suppressed the total number of small intestinal lesions and positive ratio of fecal occult blood test in this study, a placebo controlled study with large sample size is needed. Moreover, the interaction between PPIs use and egualen sodium on the NSAIDs-induced small intestinal mucosal injury should be determined.

In conclusion, in this study, the protective effect of egualen sodium on aspirin-induced small intestinal injury was shown in human subjects. Since acid suppression may cause dysbiosis and exacerbate NSAID-induced enteropathy, administration of a mucosal protective agent independent of acid suppression, such as egualen sodium, may be an additional treatment option for patients taking NSAIDs, aspirin and acid suppressants.

## Figures and Tables

**Table 1:** Subject baseline characteristics

	Control	Egualen sodium	*p*
No. subjects	9	10	
Age (year)	37.6 ± 5.5	37.3 ± 7.7	NS
BMI	25.0 ± 4.0	25.9 ± 2.9	NS
Fecal immunochemical test (ng/ml)	25.0 ± 9.3	134.8 ± 187.9	NS
Fecal transferrin (ng/ml)	8.4 ± 4.9	34.1 ± 86.8	NS
Fecal calprotectin (µg/g)	5,294.7 ± 5,489.2	9,319.2 ± 16,518.6	NS
Small bowel transit time (min)	171.0 ± 50.1	179.5 ± 96.2	NS

**Table 2:** Number of small intestinal lesions

	Control	Egualen sodium	*p*
Total No. of intestinal mucosal injuries	3.0 ± 1.1	0.9 ± 0.9	<0.01

**Table 3:** Results of noninvasive tests of intestinal damage

	Control	Egualen sodium	*p*
Fecal occult blood test (chemical), positive reaction (%)	88.9	40	0.027
Fecal immunochemical test (ng/ml)	56.6 ± 91.2	21.8 ± 3.2	NS
Fecal transferrin (ng/ml)	7.4 ± 3.7	6.7 ± 3.0	NS
Fecal calprotectin (µg/g)	13,949.8 ± 17,402.8	15,168.4 ± 26,907.5	NS
Fold increase of fecal calprotectin	5.3 ± 8.5	3.2 ± 5.7	NS

## Abbreviations

H2: histamine receptor type 2
NSAIDs: nonsteroidal anti-inflammatory drugs
PPI: proton pump inhibitor
SH: sulfhydryl
TX: thromboxane
